# Thyrotoxic periodic paralysis - a retrospective study from Southern India

**DOI:** 10.1530/ETJ-24-0164

**Published:** 2024-11-11

**Authors:** Jinson Paul, Aneez Joseph, Felix Jebasingh, Atul Ramachandra More, Julie Hephzibah, Kripa Elizabeth Cherian, Nitin Kapoor, Hesarghatta Shyamsunder Asha, Nihal Thomas

**Affiliations:** 1 Department of Endocrinology, Diabetes and Metabolism, Christian Medical College, Vellore, India; 2 Department of Critical Care, Christian Medical College, Vellore, India; 3 Department of Nuclear Medicine, Christian Medical College Vellore, India

**Keywords:** acute flaccid paralysis, Graves’ disease, periodic paralysis, thyrotoxicosis

## Abstract

**Objective:**

Thyrotoxic periodic paralysis is a rare manifestation of thyrotoxicosis. Here, we describe the clinical and biochemical features and treatment outcomes of this disorder.

**Methods:**

This retrospective study was conducted at a tertiary care centre in southern India. The clinical and biochemical features, treatment received, and therapeutic outcomes of all patients with thyrotoxicosis and acute flaccid paralysis without any other identifiable causes (cases for the study) were compared with an equal number of consecutively selected patients who presented with thyrotoxicosis but without features of paralysis (controls for the study) during the same period.

**Results:**

In total, 41 cases and controls were included in this study. The proportion of males was 92.6% and 43.9% in the cases and controls, respectively. The mean age was 32.8 (±7.6) years (cases) and 39.7 (±11.3) years (controls). In the cases, 20% of patients presented without clinical thyrotoxic features. Graves’ disease was the most common aetiology of thyrotoxicosis in both groups (92.6% of cases and 87.8% of controls). The prevalence of goitre was significantly higher among controls (90.2%) than among cases (53.7%). The mean serum potassium, free T4, total T4 and total T3 levels were significantly lower in the cases than in the controls. In these cases, two patients had an additional aetiology for persistent hypokalaemia, likely Gitelman’s syndrome.

**Conclusion:**

This is one of the largest series of thyrotoxic periodic paralysis cases in India. In subjects with thyrotoxicosis, serum potassium, free T4, total T4 and total T3 levels were significantly lower in those with periodic paralysis than in those without.

## Introduction

Graves’ disease (GD) is an autoimmune disorder that accounts for 70–80% of patients with hyperthyroidism (1, 2). The clinical manifestations of GD include atrial fibrillation, high-output cardiac failure, loss of bone mineral content, increased risk of fractures, hyper defecation, neurological manifestations, thyroid-associated ophthalmopathy, dermopathy, and acropachy. Neurological manifestations may occur in association with systemic signs of thyrotoxicosis, including headaches, cognitive impairment, movement disorders (tremors, chorea, and myoclonus), seizures, myopathy, sensory polyneuropathy, carpal tunnel syndrome, and thyrotoxic periodic paralysis (TPP) (3, 4, 5). TPP is an uncommon presentation of thyrotoxicosis, characterized by transient and recurrent episodes of flaccid muscle paralysis that affect the proximal musculature more severely than the distal musculature. TPP occurs more commonly in patients of East Asian (Japanese, Chinese, Filipino) origin, with an incidence of up to 2% among patients with hyperthyroidism when compared with 0.1–0.2% in non-Asian populations (6). Studies have shown that in the Chinese population, the prevalence of TPP is as high as 13% with hyperthyroidism (7). Autoimmune hyperthyroidism is commonly seen in females; however, the occurrence of periodic paralysis has a male preponderance and has been observed in the second and fourth decades of life. Yao showed that episodes of TPP in Chinese men are associated with elevated serum testosterone levels (8). The most common cause of TPP is GD (9). There are multiple precipitating factors that have been described in the literature, such as high-carbohydrate ingestion, strenuous exercise, upper respiratory tract infection, exposure to cold, and use of medications such as corticosteroids, acetazolamide, epinephrine, and non-steroidal anti-inflammatory medications (10). The differential diagnosis of periodic paralysis encompasses myasthenia gravis; conditions involving renal potassium wasting syndrome, Sjogren’s syndrome, Liddle, Bartter, and Gitelman’s syndrome and mitochondrial myopathies. In hypokalaemic periodic paralysis due to renal potassium wasting, hypokalaemia may persist after correction of thyrotoxicosis and hypokalaemia is evident between episodes of paralysis. The transcellular potassium shift is a key pathogenic factor in TPP. Patients with TPP may also present with cardiorespiratory symptoms (11, 12). TPP is managed with non-selective beta-blockers or rate-limiting calcium channel blockers in addition to the correction of hyperthyroidism. Early detection and treatment are of primary importance in reducing the morbidity and mortality associated with this disorder. Mutations in *CACNA1S, SCN4A, KCNJ18, KCNJ2, KCNE3, and ABCC8* have been implicated in TPP. *KCNJ18 and KCNJ2* encode rectifying potassium channels, Kir2.6, and Kir 2.1, which are regulated by thyroid hormones (13).

Although studies from China and Japan with larger sample sizes have been reported on TPP, studies describing TPP patients are limited in Indian literature, and the clinical condition of TPP appears to be less common in India when compared to Eastern Asia (14). We intended to describe the clinical and biochemical features and the treatment outcome of this disorder, in comparison to an equal number of patients with thyrotoxicosis and without paralysis.

## Materials and methods

This retrospective study was conducted in the Department of Endocrinology of a tertiary care centre in Southern India. The study period was from January 2005 to December 2019. Hospital records of patients were obtained through centralized hospital information-processing services (CHIPS). The study was approved by the ethics committee of the Christian Medical College, Vellore (Institutional Review Board approval number 13103, dated 24 June 2020). All patients who presented with acute flaccid paraparesis, quadriparesis, or with a history of documented paralysis and thyrotoxicosis (with suppressed thyroid-stimulating hormone (TSH) and elevated T4 or FT4 levels) without any other identifiable causes (cases) were compared with an equal number of consecutively selected patients who presented with thyrotoxicosis without TPP (controls) during the same time frame.

Demographic, clinical, and laboratory data of patients in both groups were collected from electronic medical records. Clinical features, including the age of onset of paralysis, precipitating factors, number of past paralytic episodes, and the extent of paralysis, were noted. Biochemical parameters, including serum potassium, bicarbonate, chloride, corrected calcium, phosphorus, magnesium, creatinine, TSH, total and free thyroid hormones, TSH receptor antibody, and anti-thyroid antibody (anti-thyroid peroxidase and anti-thyroglobulin), were collected. The details of the treatment received, duration of recovery from paralysis, need for respiratory assistance, intensive care unit admissions, and final diagnosis were documented. In addition, a definitive treatment for thyrotoxicosis, treatment outcome, and the presence of underlying secondary causes of hypokalaemia, if the serum potassium level was not normalized even after treatment for hyperthyroidism, were also documented.

The serum potassium and chloride levels were assessed using an indirect ion-selective electrode-based method. Serum bicarbonate and magnesium levels were estimated using an enzymatic-colorimetry-based method. Jaffe’s method was used to determine serum creatinine levels (Roche P 800 and Cobas 8000 chemistry analysers). TSH and total and free thyroid hormone levels were assessed using chemiluminescence immunoassay (CLIA) with a CV of 5–7% (Siemens Atellica and Siemens Centaur). TSH receptor antibody, anti-thyroid peroxidase antibody, and anti-thyroglobulin antibody levels were measured using enzyme-linked immunosorbent assay (ELISA).Data entry was performed using the EPI Data 3.1. Statistical analyses were performed using SPSS 21.0. Descriptive analyses were performed using the mean ± standard deviation (s.d.), and categorical variables were reported using frequencies and percentages. Comparisons between subjects with and without TPP were performed using two independent-sample *t*-tests.

## Results

This study included 41 patients and 41 controls. The proportion of males was 92.6 % (38/41), and the mean was 43.9 % (18/41) among the cases and controls, respectively. The clinical characteristics of the study participants are summarized in Table 1. The mean age of the cases was 32.8 (±7.6) years and that of controls was 39.7 (±11.3) years. The mean age of the cases was significantly lower than that of controls.

**Table 1:** Clinical characteristics and biochemistry of the patients. Data are presented as mean± s.d. or as *n* (%). Values in bold indicate statistical significance.

Clinical characteristics	Reference range	Thyrotoxicosis with TPP	Thyrotoxicosis without TPP	*P*
Age (years)		32.8 ± 7.6 (41)	39.7 ± 11.3 (41)	**0.002**
Male gender (%)		92.6	43.9	**0.001**
Thyrotoxic symptoms at presentation		33/41 (80.4%)	41/41 (100%)	**0.003**
GD as the cause for thyrotoxicosis		38/41 (92.6%)	36/41 (87.8%)	0.457
Presence of goitre		22/41 (53.7%)	37/41 (90.2%)	**0.001**
Ophthalmopathy		7/41 (17.1%)	11/41 (26.8%)	0.286
Thyroid dermopathy		0/41	1/41 (2.4%)	–
Acropachy		0/41	0/41	–
TSH μIU/mL	0.3–4.5	0.007 ±0.005 (41)	0.010 ± 0.030 (41)	0.494
Total T4 nmol/L	58–140	247.1 ± 82.3 (41)	281.8 ± 83.6 (41)	**0.070**
Free T4 pmol/L	11.5–22.7	47.6 ± 23.2 (41)	65.6 ± 41.2 (41)	**0.018**
Total T3 nmol/L	0.9–2.8	3.2 ± 1.3 (7)	7.3 ± 3.4 (6)	**0.012**
Potassium mmol/L	3.5–5	3.3 ± 0.9 (40)	4.3 ± 0.7 (17)	**0.001**
Sodium mmol/L	135–145	138.6 ± 3.1 (39)	137.8 ±3.2 (17)	0.399
Bicarbonate mmol/L	22–29	22.2 ± 4.3 (38)	23.4 ± 3.6 (14)	0.368
Anti-Tg Ab IU/mL (<100)				0.092
Median		27 (21)	36 (22)	
Range		9-563	6-1930	
Positive antibody, *n*		3 (14.3%)	7 (32%)	
Anti-TP Ab IU/mL (<50)				0.565
Median		193.5 (22)	271 (22)	
Range		12-689	7-950	
Positive antibody, *n*		13 (62%)	13 (59.1%)	
TSHR Ab IU/L (<1.8)		12.5± 10.8 (11)	21.0± 11.6 (23)	0.052
Positive antibody, *n*		7 (63.6%)	22 (95.7%)	

anti-Tg Ab, anti-thyroglobulin antibody; anti-TP Ab, anti-thyroid peroxidase antibody; T3, Triiodothyronine; T4,Thyroxine; TPP, Thyrotoxic periodic paralysis; TSH, thyroid-stimulating hormone; TSHR Ab, TSH receptor antibody.

Approximately two-thirds of the cases presented with quadriparesis or quadriplegia as an index presentation. The remaining patients had weakness involving only the lower limbs. Moreover, two-thirds of patients had more than one episode of paralysis. None of the cases had other neuromuscular manifestations (such as cranial nerve palsies, cerebrovascular accidents, or peripheral neuropathy). Overt clinical signs of thyrotoxicosis were seen in 33/41 (80.4%) cases, while in the controls, all had signs and symptoms of thyrotoxicosis at presentation. The prevalence of goitre was significantly higher among the controls (90.2%) than among the cases (53.7%). Ophthalmopathy was documented in 7/41 (17%) and 11/41 (26.8%) of the cases and controls, respectively. Thyroid dermopathy was observed in only one subject among the controls. None of the patients with TPP had a thyroid dermopathy. No acropachy was observed in either of the groups. There were no polyglandular endocrinopathies associated with hyperthyroidism or family history of thyrotoxicosis among the cases. In the control group, one subject had a documented family history of thyrotoxicosis. A history of precipitating events was noted in 10/41 (24.3%) cases. Glucocorticoids and acetazolamide were the most common precipitating factors noted in 10% of the patients. Other documented precipitating factors include ingestion of high-carbohydrate meals, infections, and post-thyroidectomy.

Biochemical tests at the initial presentation in both groups are shown in Table 1. The mean serum potassium level was significantly lower in cases than in controls. The mean values of free thyroxine (T4) and total triiodothyronine (T3) were significantly lower in the cases than in the controls, whereas the mean value of total T4 was not significantly different between the two groups.

Among the cases, 38/41 patients (92.6 %) had GD as the aetiology of thyrotoxicosis, and the remaining had Hashimoto’s thyroiditis in a toxic phase. In contrast, among the controls, 36/41 (87.8 %) had GD, and the remaining had toxic multinodular goitre. The median TSH receptor antibody values were 12.5 (0.31–31.6) IU/L (done in 11/41 patients) and 21.0 (1.27–40) IU/L (done in 23/41 controls) among the cases and controls, respectively. Anti-thyroglobulin antibody levels were elevated in 15% of the cases tested (20/41). However, among the tested cases, 70% had elevated anti-thyroid peroxidase antibody levels. None of the patients had clinical features of subacute thyroiditis or De-Quervain’s thyroiditis. All patients were treated with intravenous potassium correction (one ampoule containing 20 meq of potassium) until the power was 5/5, either through central or peripheral intravenous access. Once the patient takes oral feed, they all overlap with oral potassium supplements until the serum potassium level stabilizes. All patients were discharged with oral potassium supplementation.

Of the cases, 92.7% (38/41) were treated with oral propranolol, and the remaining were treated with oral verapamil. Twenty-five patients (60.9%) exclusively received anti-thyroid medication (carbimazole). Radioactive ablation using an iodine 131 (5 mCi-A standard dose at our institution) was administered to 19 (46.3%) patients (Table 2). Two patients developed TPP only after total thyroidectomy, and both had GD with large goitre and associated compressive symptoms. They were managed with freshly prepared Lugol’s iodine solution, 7–10 drops thrice daily (equivalent to 160–240 mg iodide) for 7 days prior to surgery. The heart rate was well controlled with adequate beta-adrenergic blockade. They were biochemically toxic (patient 1 free T4–45 pmol/L and patient 2 free T4–78.8 pmol/L (normal range: −11.5–22.7)) prior to the surgery. Both patients developed TPP within 24 hours in the postoperative phase and had never experienced TPP prior to thyroidectomy.

**Table 2:** Treatment received and outcome by the study patients.

	Thyrotoxicosis with TPP	Thyrotoxicosis without TPP
Treatment received		
Antithyroid medications	25/41 (60.9%)	38/41 (92.6%)
Antithyroid medications followed by RIA	20/41 (48.7%)	32/41 (78%)
Total thyroidectomy	2/41 (4.8%)	6/41 (14.6%)
Antithyroid medications alone	5/41 (12.2%)	6/41 (14.6%)
Ablation followed by antithyroid medications	2/41 (4.8%)	2/41 (4.8%)
Treatment outcome after therapy		
Median follow up	20 months	24 months
Hypothyroidism*	22/41 (53.6%)	31/41 (75.6%)
Euthyroid status after any therapy†	7/41 (17%)	8/41 (19.5%)
Persistent hyperthyroidism	2/41 (4.8%)	2/41 (4.8%)
Outcome not known	10/41 (24.3%)	0/41

*treated with levothyroxine and currently euthyroid on follow up; † not on any medications.

RIA, radioiodine ablation; TPP, thyrotoxic periodic paralysis.

Approximately a quarter of the patients (10/41) came for only a single hospital visit; hence, follow-up data and clinical outcomes were not available. Three-fourths of the patients were followed up, with a median follow-up duration of 20 months (range, 1–180 months). At the end of the median follow-up period of 20 months, 54% (22/41) had hypothyroidism and were on levothyroxine replacement, 17% (7/41) had persistent euthyroid status after therapy with either radioiodine or anti-thyroid medications, and 5% (2/41) had persistent hyperthyroidism (Table 2).

Two patients had persistent hypokalaemia even after normalization of thyroid function. These two patients were further evaluated and found to have normotensive hypokalaemia with metabolic alkalosis, hypomagnesemia, and hypocalciuria suggestive of Gitelman’s syndrome. These patients continued long-term oral potassium and magnesium supplements.

## Discussion

TPP is a rare complication that may be related to thyrotoxicosis and can be fatal if left untreated. TPP is associated with increased Na ^+^/K ^+^- ATPase activity caused by thyroid hormones, catecholamines, and insulin. Thyroid hormones can stimulate Na^+^ K^+^-ATPase in skeletal muscles via genomic and non-genomic mechanisms. Thyroid hormones act on thyroid hormone-responsive elements and upregulate transcription of the gene encoding Na^+^ K^+^-ATPase. Thyroid hormones also enhance intrinsic activity or promote membrane insertion of the pump. Excessive thyroid hormone levels may also enhance stimulation of pump activity by β2-adrenergic agonists. Insulin induces an intracellular potassium shift by stimulating the intrinsic activity or membrane insertion of Na+-K+ ATPase, causing hypokalaemia. This effect may account for the fact that a high-carbohydrate diet can be a precipitating factor of TPP (15).

This is one of the largest series of TPP on the Indian subcontinent. The proportion of thyrotoxic patients developing TPP is higher among Asian than among non-Asian populations. Some studies from East Asia have emphasized the significance of genetic variants as a reason for the higher prevalence of TPP. Loss of function mutations in the skeletal muscle-specific inward rectifying K+ (Kir) channel, particularly Kir2.6, were found to be significant in patients with TPP. These genetic variants induce a reduction in outward K+ efflux, triggering a cycle of hypokalaemia and paradoxical depolarization. This process renders Na+ channels inactive, resulting in muscle un-excitability and paralysis during TPP episodes (15). Li *et al.* identified TPP risk loci near TRIM2 and AC140912.1 (16). Rare genetic variants of TRIM2 and KCNJ2 may cause intermittent paralytic disorders; common variants adjacent to TRIM2 and KCNJ2 may regulate gene expression and, influence TPP (16). Zhao *et al.* identified two risk loci shared by TPP and Graves’ disease, highlighting TPP as a molecular subtype of GD. Their model, incorporating genetic risk scores and TPP-specific nucleotide polymorphisms, showed promise in predicting the development in patients with Graves’ (17).

In a study conducted on the Chinese population, Li *et al.* reported KCNJ18 variants in 3.1% of TPP cases. TPP patients with KCNJ18 variants had a shorter duration of paralytic attacks and a higher prevalence of muscle soreness and weakness recurrence than those without KCNJ18 variants. Thus, TPP pathogenesis involves a complex interplay between altered ion channel function and hormone activity, disruption of skeletal muscle function, and induction of paralysis. TPP episodes can occur even in the absence of hypokalaemia. It can be triggered by various factors such as infection, cold exposure, trauma, menstrual cycle, stress, glucocorticoid therapy, and alcohol consumption. Diurnal and seasonal patterns are associated with uncertain causes (18).

Our study showed a male preponderance, consistent with the findings of other studies (10, 14). Among the cases, the majority (92.7%) had GD as the aetiology for hyperthyroidism, which is not different from previous studies (14). The mean age of the study subjects with TPP was 32.8 (±7.6) years and around three-fourths were in the age group of 20–40 years. The mean age at presentation was similar to those reported in other studies conducted in India and China. One small series of 8 patients from the United States of America showed a mean age of 27 years (19). Among the cases, over 80% had clinical thyrotoxicosis while it was only less than 20% in another study (10). TPP is well known and causes recurrent periodic paralysis. In our study, one-third of patients experienced an episode of paralysis. Three-fourths of the patients had overt toxic symptoms at presentation, and more than half of them had a clinically palpable goitre. One-fourth of patients had an identifiable precipitating factor. The precipitating factors in our cohort were medication (glucocorticoids and acetazolamide), infection, carbohydrate-rich meals, and thyroidectomy. Ophthalmopathy was seen in 7/41 (17%) of patients. This was relatively higher than that reported by Chang *et al.*, who reported a 12.5% prevalence of ophthalmopathy (20). None of our patients with TPP had a family history of hyperthyroidism as opposed to a study by Chang *et al.* in which approximately 20% had a family history of hyperthyroidism (10). Gulde *et al.* identified 33 male TPP patients, with a median age of 28 years, with 85% being Hispanic. All patients in this study presented with hypokalaemia, and 23% experienced rebound hyperkalaemia post-treatment. The prevalence of TPP in patients with hyperthyroidism is approximately 0.5%. Careful potassium repletion should be advised to prevent rebound hyperkalemia (21). Careful potassium repletion should be advised, and the proportion of male sex, thyrotoxic features at presentation, and presence of goitre were significantly different between cases and controls.

The mean serum potassium level was significantly lower in the cases than in the controls. Free T4, total T4 and total T3 levels were significantly higher in the controls than in the cases. Total thyroxine levels were higher in the controls than in the cases, but the difference was not statistically significant.

Among the cases, 48.7% underwent radioiodine ablation, while among the controls 78% underwent radioiodine ablation. The proportions of study subjects who underwent total thyroidectomy were 4.8% and 14.6% among the cases and controls, respectively. Subsequently, 53.6% and 75.6% of patients had hypothyroidism among the cases and controls, respectively. In both groups, 4.8% of patients had persistent hyperthyroidism during their follow-up visits. Of these cases, one-fourth of the patients did not have a follow-up visit for which their treatment outcomes were not known. In a study by Verma *et al.*, GD was treated with anti-thyroid medications, and 11 of them underwent radioisotope ablation (14). In a prospective observational study from Taiwan, most of the patients with GD were treated with anti-thyroid medications and thyroidectomy, and oral radioactive iodine-131 ablation therapy was administered in a smaller group (10).

The retrospective nature and lack of follow-up of approximately one-fourth of the patients are significant limitations of this study. We also acknowledge the limitation that only 18 cases and controls were matched when performing propensity scoring to adjust for age and sex. TSH receptor antibody measurement was initiated on a regular basis at our centre after 2014; therefore, it was available in only 11/41 (26%) patients with TPP. We did not perform a genetic analysis of patients with TPP in our study, and we consider this a limitation in terms of interpreting the phenotypic variants that could occur in this situation.

## Conclusion

This is one of the largest series of TPP published on the Indian subcontinent. Approximately three-fourths of the participants in our study were in their third and fourth decades of life. The mean age, proportion of male patients, thyrotoxic features at presentation, and presence of goitre were significantly different between the two groups. Among the patients with thyrotoxicosis, the serum potassium level was significantly lower in those with TPP than in those without TPP. Serum free T4, total T4 and total T3 levels were significantly higher in those with TPP than in those without TPP.

## Declaration of interest

The authors declare that there is no conflict of interest that could be perceived as prejudicing the impartiality of the study reported.

## Funding

This study did not receive any specific grants from any funding agency in the public, commercial, or not-for-profit sectors.

## Author contribution statement

JP (equal data collection, writing, and statistical analysis); AJ (equal data collection and writing); FJ (data collection, review, and editing); ARM (review); JH (review); KEC (review); NK (review); HAS (review); NT (review and editing).

## Acknowledgements

We are grateful to all the patients who participated in this study. We would like to thank Dr Grace Rebecca who helped us in performing statistical analysis.
